# Impact of Initial Chest CT Findings on the Clinical Course and Hospitalization in Patients With Acute Drug Poisoning

**DOI:** 10.7759/cureus.109273

**Published:** 2026-05-20

**Authors:** Akito Kubota, Hiroyuki Kayata, Fumitaka Kato, Yasuki Nakata, Akihiro Usui, Asako Matsushima, Masanori Morita

**Affiliations:** 1 Department of Emergency and Critical Care, Nagoya City University, Graduate School of Medical Sciences, Nagoya, JPN; 2 Department of Trauma and Critical Care Medicine, Sakai City Medical Center, Sakai, JPN

**Keywords:** aspiration pneumonia, aspiration pneumonitis, chest ct scan, cost of healthcare, drug overdose and poisoning

## Abstract

Introduction: In patients with acute drug poisoning, aspiration pneumonia complicated by respiratory failure is associated with prolonged hospitalization and increased mortality. However, the association between imaging findings at presentation and subsequent clinical course has not been fully evaluated. We investigated the association between lung injury findings on initial chest computed tomography (CT)-defined as peribronchial ground-glass opacities and/or consolidation-and clinical course and health care costs in patients with acute drug poisoning.

Methods: Among 83 patients transported to our emergency department for intentional overdose or acute drug poisoning between January and August 2023, we retrospectively analyzed 57 patients who underwent chest CT at initial presentation. Patients were divided into CT-positive and CT-negative groups according to the presence of lung injury findings on initial CT. We compared patient characteristics, clinical findings, laboratory findings, clinical course, and fee-for-service costs between groups. We also performed multivariable analyses to identify factors associated with prolonged duration of intubation, prolonged length of stay, and increased medical costs.

Results: The median age was 43.5 (IQR, 27.2-57.0) years, and 17 patients were men. Lung injury findings were observed on chest CT in 16 patients (28.1%). Compared with the CT-negative group, the CT-positive group had a significantly higher proportion of patients requiring mechanical ventilation (81.2% vs 29.2%, p < 0.01), longer duration of intubation (2.0 vs 0 days, p < 0.01), longer hospital stay (6.0 vs 3.0 days, p < 0.01), and higher fee-for-service costs (Japanese yen (¥) 875,000 vs ¥487,000, p < 0.01). In multivariable analyses, CT positivity was significantly associated with prolonged duration of intubation (p < 0.01) and increased fee-for-service costs (p = 0.01).

Conclusions: Approximately one-third of patients transported for acute drug poisoning who underwent chest CT at initial presentation had lung injury findings. These CT findings were significantly associated with prolonged duration of intubation and increased fee-for-service costs. However, because this was an exploratory retrospective analysis restricted to CT-imaged patients and residual confounding is likely, the results should be interpreted cautiously. Larger studies are needed to assess external validity.

## Introduction

In Japan, the number of emergency transports for suspected medication overdose, particularly among younger individuals, has been reported to be increasing [1], and approximately 80% of these patients require hospitalization; among these hospitalized patients, 86.6% require treatment in higher-level care units, including intensive care units (ICUs) [2]. Globally, increasing health care costs related to acute drug poisoning have also been highlighted [3,4], and acute drug poisoning has become a major issue not only from social and public health standpoints but also from a health economics perspective.

In patients with acute drug poisoning, factors reported to be associated with prolonged duration of intubation include Glasgow Coma Scale (GCS) score, serum lactate concentration, and the maximum number of tablets ingested [5]. Reported risk factors for prolonged hospital stay and prolonged ICU length of stay include aspiration pneumonia, GCS score, serum lactate, serum C-reactive protein (CRP), creatine kinase, maximum number of tablets ingested, ingestion of barbiturates, and a history of psychiatric illness [5-10]. Male sex, older age, and a history of psychiatric illness have been reported as risk factors for increased health care costs [10]. Aspiration pneumonia, which has been identified as one of the risk factors for prolonged hospital stay and prolonged ICU stay, is estimated to complicate approximately 17% of acute drug poisoning cases [7]. Reported risk factors for aspiration pneumonia include intake of specific drugs such as tricyclic antidepressants, barbiturates, and opioids [6,7,9,11], as well as older age, vomiting, GCS score, and leukocyte count [6,7]. However, in these reports, “aspiration pneumonia” was defined as positive chest radiographic findings in patients with respiratory failure, irrespective of whether the process was noninfectious or infectious [6,7]. When respiratory failure is absent at presentation, concomitant lung injury may be difficult to detect clinically. Although initial chest computed tomography (CT) may reveal imaging findings suggestive of lung injury regardless of whether patients are asymptomatic or symptomatic, few studies have examined associations between such imaging findings and clinical outcomes or healthcare costs.

At our institution, when intentional overdose or acute drug poisoning is suspected, abdominal CT is performed to evaluate residual drugs or toxins in the gastrointestinal tract. During this evaluation, the scan range is extended to include the chest, and we assess for lung injury findings, defined as peribronchial ground-glass opacities and/or consolidation, as a broad category that may include chemical pneumonitis and bacterial pneumonia. In this exploratory retrospective analysis of patients with acute drug poisoning who underwent chest CT at initial presentation, we aimed to investigate the association between lung injury findings on initial chest CT and subsequent clinical course (duration of intubation and length of hospital stay) and fee-for-service costs.

## Materials and methods

We conducted a retrospective cohort study using medical records of patients aged ≥ 18 years who were transported to our emergency department with suspected intentional overdose or acute drug poisoning between January 2023 and August 2023. Patients who underwent chest CT at initial presentation were included in the analytic cohort. Eligible cases were identified using emergency transport records during the study period, and investigators reviewed electronic medical records to confirm eligibility. Patients with isolated acute ethanol intoxication due to alcohol consumption were excluded because in Japan, such cases are generally managed separately from other forms of acute drug poisoning. No other prespecified exclusion criteria were applied. Details of the ingested drugs were determined based on information obtained from witnesses or emergency medical services personnel, results of urine drug screening tests, and the number of medication blister packs found at the scene.

From the medical records, we extracted patient characteristics (age, sex, and details of ingested drugs), clinical findings at presentation (GCS score, body temperature, and presence or absence of vomiting), laboratory findings (maximum C-reactive protein level during hospitalization), and clinical course (need for intubation, duration of intubation, length of hospital stay, and fee-for-service costs). Vomiting was assessed from emergency transport records and electronic medical records, and any documented vomiting before or after arrival was classified as present. Duration of intubation and length of hospital stay were summarized in whole days; durations less than 24 hours were treated as 0 days (truncated rather than rounded). On chest CT, the presence of lung injury findings (peribronchial ground-glass opacities and/or consolidation) was classified as “CT positive,” and patients were divided into CT-positive and CT-negative groups for comparison of these variables. The presence or absence of lung injury findings was determined based on the written interpretations of the initial chest CT by the attending emergency physician or diagnostic radiologist. These interpretations were performed as part of routine clinical care, and the readers had access to clinical information; no study-specific blinding or inter-rater reliability assessment was performed.

In Japan, current guidance proposes abdominal CT to evaluate residual drugs or toxins in the gastrointestinal tract for patients with suspected oral ingestion when severe toxicity is present or clinical deterioration is anticipated [12]. At our institution, abdominal CT is performed when these conditions are met to evaluate residual gastrointestinal drug/toxin material. In addition, our institutional protocol specifies that when patients present with impaired consciousness (GCS ≤ 14 at arrival) or respiratory failure (SpO₂ < 90% on room air), CT is performed with the scan range extended to include the head and chest, irrespective of the elapsed time since ingestion, to identify causes of impaired consciousness and assess the presence of lung injury findings. Even in the absence of impaired consciousness or respiratory failure, chest and abdominal CT may be performed when the ingested substances, elapsed time since ingestion, or other clinical factors suggest potential for subsequent clinical deterioration or persistent gastric residuals. However, when the attending physician determines that these criteria are not met, omission of CT imaging is permitted.

For statistical analyses, categorical variables were summarized as counts and percentages and were compared between groups using the chi-square test; when more than 20% of cells had an expected count < 5, Fisher’s exact test was used instead. Continuous variables are expressed as medians with IQR and were compared between groups using the Mann-Whitney U test. We then used multivariable linear regression to examine associations between prespecified covariates (age, sex, vomiting, CT positivity, and GCS score at arrival) and duration of intubation, length of hospital stay, and increased fee-for-service costs. Because ventilation days showed a highly skewed distribution with many zero values, we treated ventilation duration as a continuous outcome and applied multivariable linear regression, consistent with prior studies evaluating clinical course in acute drug poisoning. A two-sided p-value < 0.05 was considered statistically significant. All analyses were performed using IBM® SPSS Statistics, version 26 (IBM Corporation, Armonk, NY, USA).

This study was conducted in accordance with the Declaration of Helsinki and was approved by the institutional ethics committee of Sakai City General Medical Center, Sakai, Japan (approval no. 23-416). In accordance with the Act on the Protection of Personal Information, patient data were anonymized. Because this was a retrospective cohort study with a past index date and a large number of eligible patients, the requirement for individual informed consent was waived.

## Results

**Figure 1:**
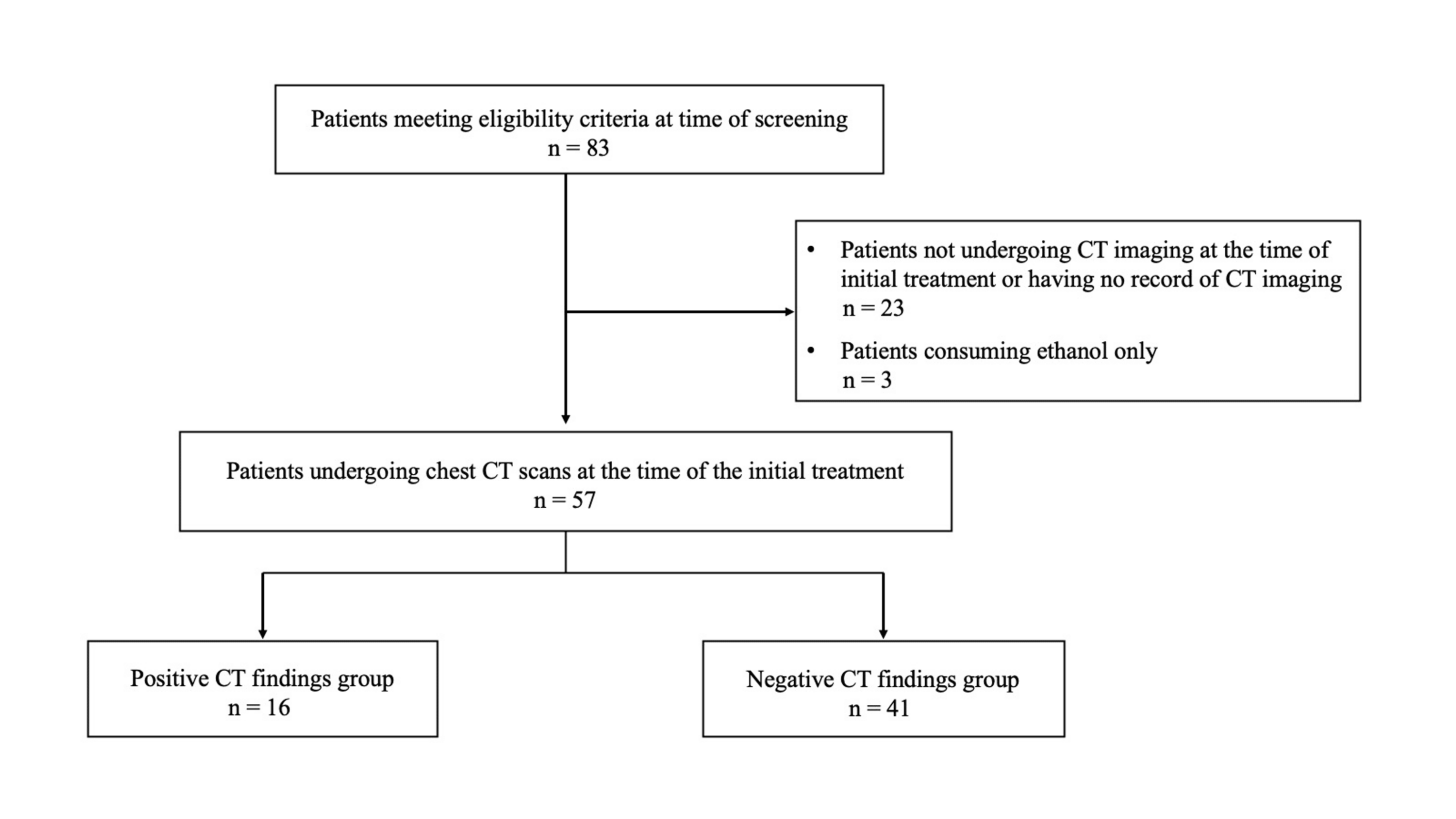
Patient recruitment flow diagram. During the study period, 83 patients were screened. Twenty-six patients were excluded according to prespecified criteria. The final analytic cohort comprised 57 patients and was classified, based on the prespecified CT definition, as CT-positive (lung injury findings present; n = 16) or CT-negative (lung injury findings absent; n = 41).

Details of patient selection are shown in Figure 1. During the study period, 83 patients were screened. Twenty-six patients were excluded: 23 did not undergo chest CT at initial presentation, and three were subsequently found to have isolated ethanol ingestion. Thus, 57 patients were included in the analysis. Among them, 16 patients (28.1%) had lung injury findings on CT, whereas 41 patients (71.9%) did not.

Details of the study cohort are presented in Table 1. The median age was 43.5 years (IQR, 27.2-57.0 years); 17 patients were men, and 40 were women. Endotracheal intubation was performed in 25 patients (43.9%); the median duration of intubation was 0 days (IQR, 0-2.0 days; durations <24 hours were treated as 0 days), the median length of hospital stay was 4.0 days (IQR, 2.0-6.0 days), and the median fee-for-service cost was Japanese yen (¥) 519,000 (IQR, ¥357,000-¥901,000), corresponding to approximately US$3,793 (IQR, US$2,609-US$6,585; January-August 2023 exchange rate: US$1 = ¥136.83).

**Table 1:** Patient characteristics. CRP: C-reactive protein; CT: computed tomography ^*^Glasgow Coma Scale ranges from 3 to 15, with lower scores indicating reduced levels of consciousness. Continuous variables are presented as medians (quartiles 1-3), and categorical variables are presented as n (%). For reference, costs were converted to U.S. dollars (US$) using the Bank of Japan Tokyo interbank US$/Japanese yen (¥) monthly average exchange rate (US$/¥ central rate, “average in the month”) averaged over the study period (January–August 2023; US$1 = ¥136.83).

Variables	Overall
	n = 57
Demographic characteristics	
Age (years)	43.5 (27.2-57.0)
Males	17 (29.8)
Clinical characteristics on admission	
Glasgow Coma Scale*	12 (7-14)
Body temperature (℃)	36.6 (36.6-37.0)
Presence of vomiting	13 (22.8)
Maximum CRP value during hospitalization (mg/dL)	0.86 (0.08-3.53)
Presence of lung injury findings on initial chest CT	16 (28.1)
Intubation	25 (43.9)
Days of mechanical ventilation	0 (0-2.0)
Days in hospital	4.0 (2.0-6.0)
Cost of hospitalizations-×10^4^ (¥)	51.9 (35.7-90.1)

Comparisons between the CT-positive and CT-negative groups are shown in Table 2. There were no significant between-group differences in patient characteristics (age, sex, and ingested drugs) or clinical findings at presentation (GCS score, body temperature, and presence or absence of vomiting). The ingested drugs were recorded on the basis of information obtained from witnesses or emergency medical services personnel, and the results of urine drug screening tests, and cases involving the ingestion of multiple drugs were counted in more than one category. The ingested dose was not recorded because accurate quantification was not feasible. The maximum CRP level was significantly higher in the CT-positive group than in the CT-negative group (13.02 mg/dL vs 0.22 mg/dL; p < 0.01). The CT-positive group also had a significantly higher rate of intubation (81.2% vs 29.2%; p < 0.01), longer duration of intubation (2.0 days vs 0 days; p < 0.01), longer hospital stay (6.0 days vs 3.0 days; p < 0.01), and higher fee-for-service costs (¥875,000 vs ¥487,000; approximately $6,395 vs $3,560; p < 0.01) than the CT-negative group.

**Table 2:** Patient characteristics by lung injury findings on initial chest CT (peribronchial ground-glass opacities and/or consolidation). CT: computed tomography; OTC: over the counter; SSRI: selective serotonin reuptake inhibitors; SNRI: serotonin-norepinephrine reuptake inhibitors; CRP: C-reactive protein. ^*^Any sedative/hypnotic agent was defined as the presence of any of the following drug categories: benzodiazepines, tricyclic antidepressants, antipsychotics, mood stabilizers, barbiturates, or opioids. ^†^Glasgow Coma Scale ranges from 3 to 15, with lower scores indicating reduced levels of consciousness. Categorical variables are presented as numbers and percentages. Group comparisons were performed using chi-square tests and Fisher’s exact tests when more than 20% of expected frequencies were below five. Continuous variables are presented as medians [quartiles 1-3], and group comparisons were performed using the Mann-Whitney U test. Statistical significance was set at p < 0.05 for group comparison. Categories are not mutually exclusive; therefore, percentages are calculated using the group denominator (n = 16 or n = 41), and totals may exceed 100%. P-values for individual drug categories are not reported due to sparse data. For reference, costs were converted to U.S. dollars (US$) using the Bank of Japan Tokyo interbank US$/Japanese yen (¥) monthly average exchange rate (US$/¥ central rate, “average in the month”) averaged over the study period (January–August 2023; US$1 = ¥136.83).

Variables	Lung injury findings on initial chest CT		
	Positive	Negative	
	n = 16	n = 41	p
Demographic characteristics			
Age (years)	54 (22.2-59.5)	42 (25.0-52.0)	0.114
Male (sex)	5 (31)	12 (29.2)	1
Type of drug, including overdose drugs			
Benzodiazepine	8 (50.0)	23 (56.0)	
OTC drugs	3 (18.8)	11 (26.8)	
Ethanol	1 (6.3)	6 (14.6)	
Mood stabilizers	2 (12.5)	6 (14.6)	
Antipsychotics	2 (12.5)	5(12.1)	
SSRI/SNRI	0 (0)	3 (7.3)	
Tricyclic antidepressants	3 (18.8)	1 (2.4)	
Barbiturates	0 (0)	0 (0)	
Opioids	0 (0)	2 (4.9)	
Others	5 (31.3)	7 (17.0)	
Unknown	0 (0)	1 (2.4)	
Number of drug classes per patient	2 [1-3]	2 [1-3]	0.583
Any sedative/hypnotic agent*	9 (56.3)	31 (75.6)	0.201
Clinical characteristics on admission			
Glasgow Coma Scale†	7 (6-12)	12 (9-14)	0.24
Body temperature (℃)	36.6 (35.9-37.4)	36.6 (36.1-37.0)	0.844
Presence of vomiting	5 (31)	8 (19.5)	0.479
Maximum CRP value during hospitalization (mg/dL)	13.02 (2.10-19.44)	0.22 (0.07-1.63)	<0.01
Intubation	13 (81.2)	12 (29.2)	<0.01
Days of mechanical ventilation	2.0 (0.5-4.0)	0 (0-2.0)	<0.01
Days in hospital	6.0 (4.0-11.5)	3.0 (2.0-5.0)	<0.01
Cost of hospitalizations-×10^4^ (¥)	87.5 (59.7-153.7)	48.7 (33.5-65.6)	<0.01

In multivariable analyses of risk factors for prolonged duration of intubation, prolonged hospital stay, and increased fee-for-service costs in the overall cohort, CT positivity at initial presentation was significantly associated with prolonged duration of intubation (p < 0.01) and increased fee-for-service costs (p = 0.01). In contrast, no variables were significantly associated with prolonged hospital stay (Tables 3-5).

**Table 3:** Factor analysis of prolonged ventilation time using multiple regression analysis. CI: confidence interval; CT: computed tomography Adjusted R² = 0.21, overall model F-test p < 0.05, two-sided tests: α = 0.05; multivariable linear regression was used to identify independent predictors of prolonged ventilation time.

Variables	Standardized estimate (β)	Difference (95% CI)	p
Age	0.11	-0.02 to 0.50	0.39
Male sex	-0.1	-1.87 to 0.78	0.41
Presence of vomiting	0.14	-0.68 to 2.27	0.28
Presence of lung injury findings on initial chest CT	0.4	0.61 to 3.78	<0.01
Glasgow Coma Scale score	-0.13	-0.28 to 0.10	0.35

**Table 4:** Factor analysis of prolonged hospitalization using multiple regression analysis. CI: confidence interval; CT: computed tomography. Adjusted R² = 0.13; overall model F-test p < 0.05; two-sided tests: α = 0.05; multivariable linear regression was used to identify independent predictors of prolonged hospitalization.

Variables	Standardized estimate (β)	Difference (95%CI)	p-value
Age	0.19	-0.03 to 0.18	0.17
Male sex	-0.21	-7.66 to 0.75	0.10
Presence of vomiting	0.12	-2.60 to 6.49	0.39
Presence of lung injury findings on initial chest CT	0.29	-0.11 to 9.40	0.05
Glasgow Coma Scale score	0.08	-0.42 to 0.75	0.58

**Table 5:** Factor analysis of increased hospitalization costs using multiple regression analysis. CI: confidence interval; CT: computed tomography. Adjusted R² = 0.19; overall model F-test p = 0.05; two-sided tests: α = 0.05; multivariable linear regression was used to identify independent predictors of increased hospitalization costs.

Variables	Standardized estimate (β)	Difference (95% CI)	p-value
Age	0.11	-6213 to 16577	0.36
Male sex	-0.28	-977744 to -42608	0.03
Presence of vomiting	0.11	-286652 to 726077	0.38
Presence of lung injury findings on initial chest CT	0.37	145065 to 1134755	0.01
Glasgow Coma Scale score	0.1	-41948 to 84400	0.50

## Discussion

In this study, approximately one-third of patients transported for acute drug poisoning had lung injury findings, peribronchial ground-glass opacities and/or consolidation, on chest CT at initial presentation, irrespective of the presence of respiratory failure. Compared with patients without such findings, those with CT-positive lung injury findings had significantly higher rates of endotracheal intubation, longer duration of intubation, longer hospital stay, and higher fee-for-service costs. Furthermore, regardless of respiratory failure status, CT positivity at presentation was significantly associated with prolonged duration of intubation and increased fee-for-service costs.

In previous studies reporting an impact of aspiration pneumonia on hospital length of stay and ICU length of stay [6,7], the pneumonia group was defined as patients with positive findings on chest radiography in addition to respiratory failure. In the present study, by contrast, we defined CT positivity as lung injury findings, peribronchial ground-glass opacities, and/or consolidation on initial chest CT. On the basis of prior reports that chest CT has a higher detection rate for pulmonary parenchymal abnormalities than plain chest radiography [13-15], we used these CT findings as a pragmatic definition of lung injury. In addition, we evaluated CT findings irrespective of the presence of respiratory failure, which distinguishes our study from prior reports on acute drug poisoning and aspiration-related pulmonary complications. In our cohort, the presence of lung injury findings on initial chest CT was associated with subsequent prolongation of intubation and increased fee-for-service costs. These findings may serve as a prognostic marker reflecting overall illness severity at presentation. However, because we did not evaluate respiratory status at presentation in detail, whether lung injury findings are associated with respiratory failure or other measures of respiratory status warrants further investigation. Moreover, this was a small retrospective study, and the decision to perform CT and subsequent management was at the discretion of the attending physician; thus, confounding and selection bias may have influenced these associations.

In the emergency setting, CT can provide substantial diagnostic information within a short time and is highly useful for the timely diagnosis of a broad range of conditions, including life-threatening diseases, in appropriately selected patients. Based on our findings, risk stratification using initial chest CT findings may be considered, and, as a hypothesis, CT-positive patients could be managed with closer respiratory monitoring, a lower threshold for ICU admission, and repeat evaluation when clinically indicated. However, excessive imaging entails disadvantages such as radiation exposure, increased medical costs, prolonged emergency department length of stay, and the need to address incidental findings. Therefore, imaging stewardship using clinical decision tools has been proposed in recent years. For example, the Falls decision rule in older adults with ground-level falls may reduce head CT use by approximately one-third while maintaining high sensitivity and negative predictive value [16]. Future prospective studies will be needed to determine whether performing chest CT at presentation and/or CT-informed risk stratification and management directly influence outcomes in specific patient subgroups.

Although the type and dose of ingested drugs are used as predictors of severity in acute drug poisoning [5,8,11,17], this information is generally based on reports from the patient or witnesses and on the number of medication blister packs found by emergency medical services. Such information is not necessarily accurate, and it is often difficult to infer the exact drugs and doses ingested. Urine drug screening does not detect all possible substances, and emergent measurement of serum drug concentrations is available for only a limited number of agents; therefore, these variables were not included in the present analysis.

In contrast to prior reports identifying GCS score as a risk factor for prolonged duration of intubation and prolonged hospital stay, GCS score was not a significant factor in our study. One possible explanation is that poisoning due to long-half-life barbiturates has decreased as these drugs have become more strictly regulated, leading to fewer such cases [18]. Further, there are substantial epidemiologic differences in causative drugs between Japan and other countries. For example, in Japan, the number of hospitalizations for opioid poisoning was 17.0 per 100,000 population in 2016 [19], whereas in the United States, the corresponding figure was 295.6 per 100,000 population in 2012 [20]. These differences in the epidemiology of causative agents may have influenced our results.

Chest CT findings were also not identified as risk factors for a prolonged hospital stay. In the management of acute drug poisoning, in addition to somatic treatment, psychiatric intervention and social support are often required, and many patients cannot be discharged immediately once their physical condition has stabilized. Previous studies have reported that a history of psychiatric illness prolongs hospital stay, possibly because psychiatric assessment and arrangements for psychiatric admission require additional time [10]. At our institution, once physical problems have been resolved, a liaison team comprising psychiatrists and specialized nurses evaluates whether continued psychiatric hospitalization is necessary, and when indicated, the team coordinates transfer to an affiliated psychiatric hospital or another facility capable of providing inpatient care. Even for patients who do not require continued hospitalization, we arrange ongoing treatment with their primary psychiatrist or another mental health provider to prevent recurrence of overdose or self-harm. In the inpatient care of patients with acute drug poisoning, the duration of psychiatric and social coordination may, in some cases, determine the period of healthcare resource use more than the duration of intubation or intensive care. This may have influenced the analysis of risk factors for prolonged hospital stay.

Among aspiration-related pulmonary disorders, two disease entities are encompassed within the definition of pneumonia arising from aspiration: aspiration pneumonitis and aspiration pneumonia. The former is characterized predominantly by direct injury to the lung parenchyma and subsequent chemical pneumonitis caused by aspiration of gastric contents, whereas the latter is characterized mainly by bacterial pneumonia resulting from aspiration of bacteria and saliva present in the oropharynx [21,22]. In the context of acute drug poisoning, the clinical significance of these entities differs: aspiration pneumonitis suggests aspiration of acidic or otherwise highly cytotoxic material, whereas aspiration pneumonia suggests prolonged impairment of consciousness to the extent that pneumonia can develop. Another clinically important entity that does not neatly fit into either category is acute respiratory distress syndrome secondary to inhalation of toxic gases, in addition to the implicated ingested substance [23]. In many cases, it is difficult to differentiate among these entities based solely on chest CT findings, and this limitation must be considered when determining treatment strategies.

The principal limitation of this study is that it was a single-center retrospective cohort study with a small sample size. In addition, chest CT acquisition was at the discretion of the attending physician, and more severely ill patients may have been more likely to undergo chest CT, introducing potential confounding and selection bias. Therefore, based on this study alone, the presence or absence of CT findings may represent a surrogate marker of baseline severity and aspiration burden rather than an independent prognostic factor. Moreover, because our analysis was restricted to patients who underwent chest CT, the findings cannot be generalized to all patients with acute drug poisoning. Clinical decision-making, including intubation and intensive care management, was not based on a research-standardized protocol and cannot be defined in a fully reproducible manner from retrospective documentation. Finally, lung injury findings on chest CT were determined from routine clinical interpretation reports, and the readers were not blinded to clinical information, which may have introduced observer bias.

Several clinically important confounders were not measured or not included in the analysis, such as baseline pulmonary comorbidities, immunocompromised status, witnessed aspiration status, oxygen requirement at presentation, serum lactate levels, the specific types and doses of ingested drugs, and the time interval from ingestion to imaging. These unmeasured factors may have influenced both CT findings and clinical outcomes. In particular, because respiratory status at presentation (SpO₂, oxygen supplementation, respiratory rate, arterial blood gas findings, and the presence of respiratory failure) was not systematically collected or analyzed, we cannot determine whether lung injury findings on chest CT provide prognostic information independent of baseline respiratory compromise at presentation.

The distributions of ventilation days and hospitalization costs were highly skewed, and ventilation duration included many zero values. Although we analyzed these outcomes as continuous variables using multivariable linear regression, this approach may have limited power and stability. No robust regression techniques or formal diagnostic checks beyond inspection of residual patterns were performed, and these factors should be considered when interpreting the results.

Finally, aspiration pneumonitis (theoretically noninfectious), aspiration pneumonia (bacterial), non-aspiration-related infectious pneumonia, and dependent atelectasis may all have been present in our cohort, and the relationship between the presence of CT findings and the effectiveness of antimicrobial therapy remains unclear. Further studies with larger cohorts, adjustment for patient background characteristics, and prospective designs are warranted.

## Conclusions

Approximately one-third of the study patients transported to our emergency department with acute drug poisoning who underwent chest CT at initial presentation had peribronchial ground-glass opacities and/or consolidation on chest CT. These CT findings were significantly associated with prolonged duration of intubation and increased in-hospital costs. However, because this was an exploratory retrospective analysis restricted to CT-imaged patients and residual confounding is likely, the results should be interpreted cautiously. Larger studies are needed to assess external validity.
